# Comparison of intravitreal anti-VEGF agents and oral carbonic anhydrase inhibitors in the treatment of cystoid macular edema secondary to retinitis pigmentosa

**DOI:** 10.3389/fphar.2024.1477889

**Published:** 2024-12-10

**Authors:** Jia Liang, Xueping Wu, Lu Chen, Lujia Feng, Xiangqing Hei, Yingying Diao, Yuke Ji, Huiyan Zheng, Zhenhua Zou, Dong Fang, Shaochong Zhang

**Affiliations:** ^1^ Shenzhen Eye Hospital, Shenzhen Eye Institute, Shenzhen, Guangdong, China; ^2^ Xiamen Eye Center and Eye Institute of Xiamen University, Xiamen, China

**Keywords:** anti-vegf, carbonic anhydrase inhibitors, cystoid macular edema, retinitis pigmentosa, clinical efficacy

## Abstract

**Purpose:**

To compare the efficacy of intravitreal antivascular endothelial growth factor (anti-VEGF) agents with oral carbonic anhydrase inhibitors (CAIs) in treating cystoid macular edema (CME) secondary to retinitis pigmentosa (RP).

**Methods:**

This retrospective study analyzed 98 patients (98 eyes) with RP-CME: 47 (48.0%) received intravitreal anti-VEGF agents (Ranibizumab or Bevacizumab) and 51 (52.0%) were treated with oral CAIs (methazolamide 50 mg/day or acetazolamide 500 mg/day). Medical records were reviewed to assess best-corrected visual acuity (BCVA), central macular thickness (CMT), and intraocular pressure (IOP) at baseline and at 1, 3, 6, and 12 months post-treatment using Generalized Estimation Equations (GEE). Adverse events and risk factors influencing visual prognosis were also evaluated.

**Results:**

Both groups showed significant improvement in BCVA and reduction in CMT at 1 and 3 months post-treatment compared to baseline (all *p* < 0.001). In the oral CAIs group, these improvements persisted until 6 months. However, by 12 months, neither group exhibited significant improvements in BCVA or CMT compared to baseline (all *p* > 0.05). Intragroup comparisons revealed that the oral CAIs group had significantly better BCVA and CMT improvements at 3 and 6 months than intravitreal anti-VEGF group (*p* < 0.001 for BCVA at 3 months, *p* = 0.003 for BCVA at 6 months; all *p* < 0.001 for CMT at both 3 and 6 months). No significant differences were found between the two groups in BCVA and CMT at 12 months or in IOP at any time point (all *p* > 0.05). Subgroup analysis indicated that oral acetazolamide was more effective than methazolamide in reducing CMT and improving BCVA at 3 and 6 months (*p* = 0.005 for BCVA at 3 months, *p* = 0.015 for BCVA at 6 months; *p* = 0.037 for CMT at 3 months, *p* < 0.001 for CMT at 6 months). There were no significant differences in outcomes between intravitreal Ranibizumab and Bevacizumab (all *p* > 0.05). Correlation analysis showed that worse BCVA at 12 months was associated with older age (r = 0.202, *p* = 0.046), higher baseline CMT (r = 0.353, *p* < 0.001), poorer baseline BCVA (r = 0.579, *p* < 0.001), but showed no correlation with genotype. Adverse effects from oral CAIs included tingling sensation (3.9%), altered taste (9.8%), and gastrointestinal upset (7.8%). The Ranibizumab group required an average of 3.7 ± 0.8 treatments, and the Bevacizumab group required an average of 3.8 ± 0.5 treatments over the course of 1 year without experiencing severe adverse effects.

**Conclusion:**

Both intravitreal anti-VEGF agents and oral CAIs effectively improved CMT and BCVA in RP-CME patients within the first 3 months of treatment. However, oral CAIs demonstrated superior anatomic and functional improvements at 6 months. Poorer BCVA prognosis was associated with older age, higher baseline CMT, poorer baseline visual acuity. Larger, randomized clinical trials with extended follow-up periods are needed to confirm these findings.

## Introduction

Retinitis pigmentosa (RP) is a common inherited retinal dystrophy caused by gene mutations (Ferrari et al., 2011). It is characterized by symptoms such as nyctalopia, followed by peripheral visual field loss and eventual central vision impairment (Bakthavatchalam et al., 2018). Cystoid macular edema (CME) is a recognized complication of RP, with an incidence ranging from 10% to 50%, significantly contributing to significant central visual impairment in affected patients (Liew et al., 2019; Grigoropoulos et al., 2010; Sandberg et al., 2008; Testa et al., 2014). While the pathogenesis of CME secondary to RP remains poorly understood, hypotheses suggest the involvement of macular Müller cell injury, autoimmune phenomena, breakdown of the blood-aqueous barrier, and/or chronic inflammatory reactions (Kim, 2006; Strong et al., 2017; Yoshida et al., 2013).

Optical coherence tomography (OCT) has become a standard tool for diagnosing and monitoring CME, providing crucial morphological insights for treatment and follow-up (Arrigo et al., 2023). Central Macular Thickness (CMT) is used to assess the severity of CME and is typically closely related to the patient’s visual function. Current therapeutic approaches for CME associated with RP include oral/topical carbonic anhydrase inhibitors (CAIs), antivascular endothelial growth factor (anti-VEGF) agents, focal laser therapy, internal limiting membrane peeling surgery and intravitreal steroids. Although these methods have been reported to be effective in treating RP-CME, their mechanisms of action differ. For example, anti-VEGF treatments (e.g., Ranibizumab and Bevacizumab) work by inhibiting the activity of VEGF, thereby preventing pathological angiogenesis and vascular leakage, which reduces fluid leakage into the retina and subretinal space. On the other hand, oral CAIs (including methazolamide and acetazolamide) inhibit the enzyme carbonic anhydrase within the eye, which is involved in the production of bicarbonate ions by the ciliary body. CAIs inhibition reduces the production of aqueous humor and lowers intraocular pressure, thereby indirectly relieving macular edema.

Despite the expanding treatment options for CME in RP, there remains a paucity of comparative studies evaluating the efficacy of different treatments or determining optimal regimens. Thus, there is a critical need to establish effective treatments for CME associated with RP. In this study, we aim to compare the anatomical and functional outcomes of intravitreal anti-VEGF agents and oral CAIs, evaluating their efficacy, duration of action, and complications in treating CME in RP eyes. Comparing the efficacy of these two approaches will also allow for an evaluation of the benefits and drawbacks of directly inhibiting neovascularization versus modulating intraocular fluid balance, providing new insights into the pathological mechanisms and treatment of RP-CME.

## Methods

### Study subjects

This retrospective cohort study included 98 patients (98 eyes) diagnosed with CME secondary to RP, who received either oral CAIs (30 methazolamide and 21 acetazolamide) or intravitreal anti-VEGF agents (29 Ranibizumab or 18 Bevacizumab) between July 2008 and May 2023. This study was approved by the Ethics Committee of the Shenzhen Eye Hospital, Shenzhen, China, and conformed to the principles of the Declaration of Helsinki. Inclusion criteria encompassed: (1) confirmed RP-CME diagnosis; (2) completion of a 12-month follow-up. Exclusion criteria included: (1) macular complications, such as epiretinal membrane and vitreous traction, macular atrophy or other causes of poor fixation and loss of vision; (2) other diseases, such as diabetic retinopathy or glaucoma; (3) previous ocular surgery except uneventful cataract surgery performed within 12 months before study entry; (4) intolerance to CAIs or pre-existing electrolyte imbalances or abnormal liver and kidney function. (5) other treatments for RP-CME, such as intravitreal injection of triamcinolone or other therapies.

### Data collection

Data retrieved from the medical records included demographic information, medical history, history of ocular conditions, and previous treatments for RP. All participants underwent detailed ophthalmic examinations before treatment and at 1, 3, 6, and 12 months post-treatment, including the best-corrected visual acuity (BCVA) test, represented as logarithm of the minimum angle of resolution (logMAR), slit-lamp examination, refractive status assessment using an autorefractor and active optometry (KR-8800, Topcon, Tokyo, 69 Japan); Central macular thickness (CMT) analyzed by the spectral-domain optical coherence tomography (OCT) (Spectralis HRA + OCT; Heidelberg Engineering, Inc., Heidelberg, Germany). CMT refers to a retinal thickness within the central 1.0 mm on OCT B-scans and measurements were jointly performed by two experts (SCZ and LC). Intraocular pressure (IOP) was measured using a non-contact tonometer (Topcon, Tokyo, Japan), with the normal range being 10–21 mmHg. Complications and adverse effects were documented and interventions results.

### Treatment protocol

Patients in the anti-VEGF group received intravitreal injections of anti-VEGF agents at baseline, administered 3.5–4.0 mm from the limbus. Macular edema recurrence was defined as an increase in CMT by more than 30 μm from the post-treatment lowest value, accompanied by a decrease in visual acuity of more than 5 ETDRS letters (Lee et al., 2021; Jang et al., 2020). Retreatment was permitted if CMT exceeded 350 mm (Al Qassimi et al., 2022). Patients in oral CAIs group treated with methazolamide (50 mg/day) or acetazolamide (500 mg/day).

### Statistical considerations

Comparative analyses of categorical variables were conducted using the chi-square test. Normally distributed data are presented as mean ± standard deviation (SD) and were compared using independent sample t-tests. Changes in BCVA, CMT, and IOP at different time points were analyzed using Generalized estimation equations (GEE) The GEE method is particularly suitable for analyzing correlated or repeated measures data, as it accounts for the correlation between observations within the same group, which standard linear regression cannot address. Key confounding factors, such as patient age, disease duration, inheritance pattern, and disease severity, were included as covariates in the GEE analysis to exclude the interference. The correlation between BCVA prognosis and other factors was assessed using Pearson correlation analysis. Statistical analysis was performed using SPSS 22.0 (IBM, Armonk, NY, United States), with *p* < 0.05 considered statistically significant.

## Results

### Clinical characteristics of eyes with RP-CME

A total of 98 eyes from 98 patients with CME secondary to RP were included in the analysis, consisting of 52 women and 46 men, with a mean age of 46.06 ± 13.87 years (range 18.0–76.0). The study group comprised 47 patients (47.96%) receiving intravitreal anti-VEGF agents including Ranibizumab (61.70%) and Bevacizumab (38.30%), and 51 patients (52.04%) treated with oral CAIs including methazolamide (58.82%) and acetazolamide (41.18%). Baseline characteristics between the two groups did not exhibit statistically significant differences (all *p* > 0.05) (Table 1). Among patients receiving anti-VEGF agents, 55.32% had autosomal dominant RP, 42.55% had autosomal recessive RP, and 2.13% had X-linked RP. Of those receiving CAIs, 54.90% had autosomal dominant RP, 43.14% had autosomal recessive RP, and 2.13% had X-linked RP (Table 1).

**TABLE 1:** Baseline characteristics of study participants.

Characteristics	Anti-VEGF agents (n = 47)	Oral CAIs (n = 51)	*P*-value
Sex, female: male	29:18	23:28	0.100
Age (years); range	47.45 ± 13.86 18–76	44.78 ± 13.89 22–75	0.345
Duration of RP (years); range	10.31 ± 3.01 4–17	10.65 ± 3.41 4.5–17	0.605
CMT (mm), range	431.53 ± 67.55 324–620	427.55 ± 90.67 303–657	0.802
BCVA (logMAR); range	0.64 ± 0.24 0.40–1.30	0.67 ± 0.23 0.30–1.30	0.497
IOP (mmHg), Mean (SD); range	12.75 ± 1.47 10.20–16.70	13.09 ± 1.94 9.80–18.30	0.322
Phakic eyes, n (%)	20	23	0.800
Retinitis pigmentosa, (%)			
Autosomal dominant, n (%)	26 (55.32)	28 (54.90)	0.873
RHO	8	6	
NRL	2	3	
RP1	1	1	
RP9	1	1	
PRPF31	2	1	
IMPDH1	0	2	
Unknown/not tested	12	14	
Autosomal recessive, n (%)	20 (42.55)	22 (43.14)	0.862
CRB1	3	1	
USH2A	2	2	
Unknown/not tested	15	19	
X-linked	1 (2.13)	1 (2.13)	1.000
Unknown/not tested	1	1	1.000

Autosomal dominant, autosomal recessive, and X-linked were determined clinically, with molecular confirmation in some patients. CAI, carbonic anhydrase inhibitor; CMT, central macular thickness; BCVA, best-corrected visual acuity; logMAR, logarithm of the minimum angle of resolution; IOP, intraocular pressure.

Data are presented as the means ± SD.

Bold values indicate statistical significance *P* < 0.05.

### Intergroup evaluation in anti-VEGF and oral CAIs groups in RP-CME

**FIGURE 1:**
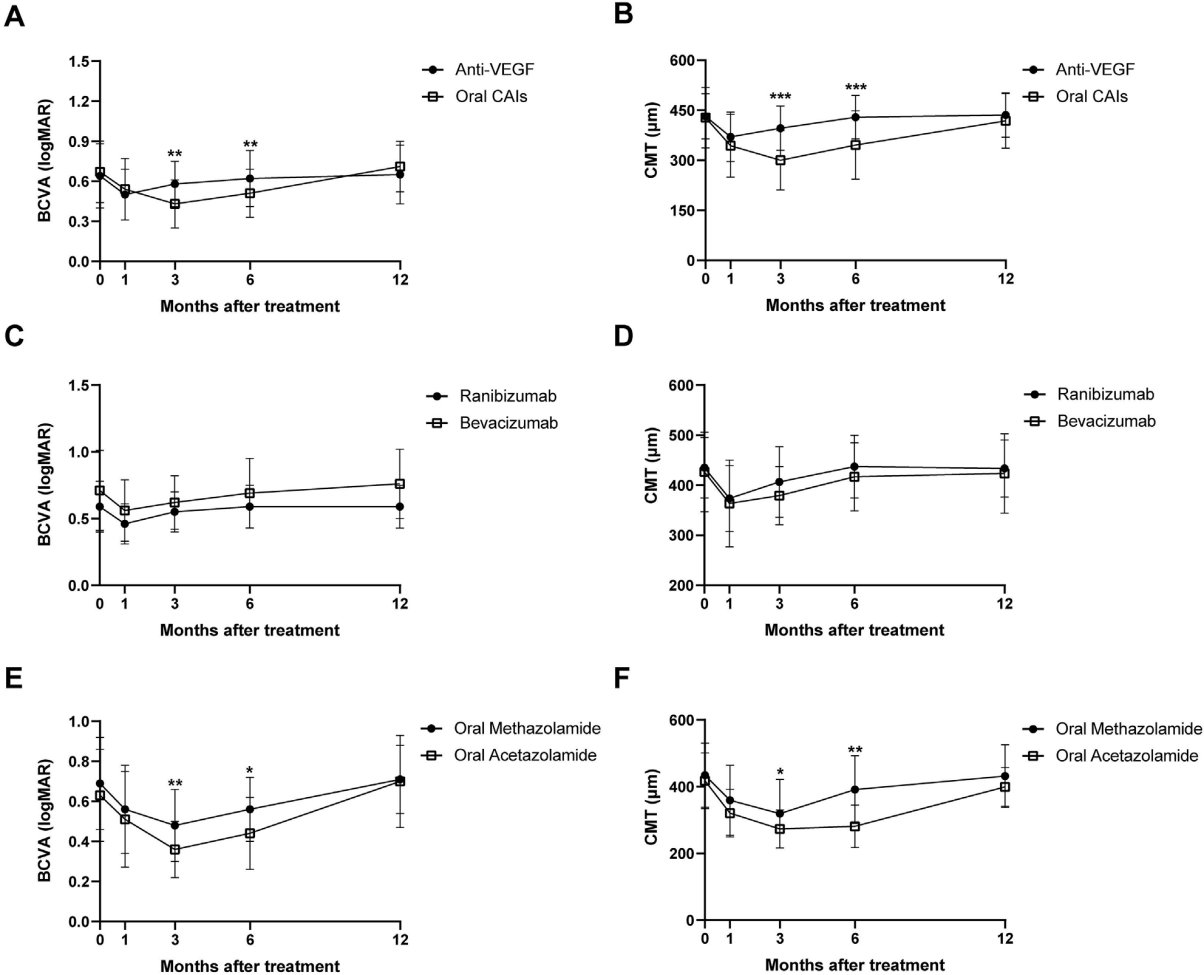
Changes in BCVA **(A)** and CMT **(B)** over 12 months for anti-VEGF and oral CAIs using Generalized Estimating Equations. Changes in BCVA **(C)** and CMT **(D)** over 12 months for intravitreal Ranibizumab and intravitreal Bevacizumab. Changes in BCVA **(E)** and CMT **(F)** over 12 months for oral Methazolamide and oral Acetazolamide. BCVA, best corrected visual acuity; CMT, central macular thickness; CAI, carbonic anhydrase inhibitors.

Patients treated with anti-VEGF showed BCVA improvements from 0.64 ± 0.24 logMAR at baseline to 0.5 ± 0.19 logMAR at month 1 (*p* < 0.001) and 0.58 ± 0.17 logMAR at month 3 (*p* < 0.001), with no significant changes at months 6 and 12 (all *p* > 0.05). Conversely, the oral CAIs group improved from 0.67 ± 0.23 logMAR at baseline to 0.54 ± 0.23 logMAR at month 1, 0.43 ± 0.18 logMAR at month 3, and 0.51 ± 0.18 logMAR at month 6 (all *p* < 0.001), but no significant change at month 12 (*p* = 0.061). For CMT, in the anti-VEGF group, CMT decreased significantly from 431.53 ± 67.55 μm at baseline to 370.30 ± 73.85 μm at month 1 and 396.11 ± 66.82 μm at month 3 (all *p* < 0.001), with no significant changes at months 6 and 12 (all *p* > 0.05). Similarly, the oral CAIs group showed reductions in CMT from 427.55 ± 90.67 μm at baseline to 343.57 ± 94.09 μm at month 1, 300.10 ± 89.17 μm at month 3, and 345.94 ± 102.94 μm at month 6 (all *p* < 0.001), with no significant change at month 12 (*p* = 0.150) (Table 2; Supplementary Table S1; Figure 1).

**TABLE 2:** Intragroup comparisons of visual acuity, central macular thickness and intraocular pressure with baseline in two groups using Generalized estimating equations.

Group	Characteristics	BCVA (logMAR)	95%CI	*P*-value	CMT (μm)	95%CI	*P*-value	IOP (mmHg)	95%CI	*P*-value
Anti-VEGF (n = 47)	Baseline	0.64 ± 0.24			431.53 ± 67.55			12.75 ± 1.47		
	Month 1	0.50 ± 0.19	[−0.161, −0.112]	<**0.001**	370.30 ± 73.85	[−75.497, −46.971]	**<0.001**	12.73 ± 1.49	[−0.144, 0.101]	0.734
	Month 3	0.58 ± 0.17	[−0.084, −0.030]	**<0.001**	396.11 ± 66.82	[−49.533, −21.318]	**<0.001**	12.77 ± 1.49	[−0.122, 0.169]	0.752
	Month 6	0.62 ± 0.21	[−0.032, 0.010]	0.302	429.34 ± 64.92	[−11.138, 6.755]	0.631	13.71 ± 1.40	[−0.169, 0.092]	0.564
	Month 12	0.65 ± 0.22	[−0.027, 0.062]	0.440	435.81 ± 66.48	[−4.013, 12.566]	0.312	12.74 ± 1.36	[−0.136, 0.128]	0.950
Oral CAIs (n = 51)	Baseline	0.67 ± 0.23			427.55 ± 90.67			13.09 ± 1.94		
	Month 1	0.54 ± 0.23	[−0.150, −0.105]	**<0.001**	343.57 ± 94.09	[−100.330, −67.631]	**<0.001**	13.03 ± 1.99	[−0.190, 0.081]	0.427
	Month 3	0.43 ± 0.18	[−0.278, −0.193]	**<0.001**	300.10 ± 89.17	[−148.966, −105.936]	**<0.001**	13.17 ± 2.00	[−0.047, 0.216]	0.208
	Month 6	0.51 ± 0.18	[−0.204, −0.113]	**<0.001**	345.94 ± 102.94	[−101.838, −61.377]	**<0.001**	13.17 ± 2.02	[−0.062, 0.231]	0.260
	Month 12	0.71 ± 0.19	[−0.027, 0.104]	0.250	418.29 ± 82.05	[−21.849, 3.339]	0.150	13.11 ± 1.97	[−0.125, 0.165]	0.791

CMT, central macular thickness; BCVA, best-corrected visual acuity; logMAR, logarithm of the minimum angle of resolution; IOP, intraocular pressure.

Data are presented as the means ± SD.

Bold values indicate statistical significance *P* < 0.05.

### Intragroup comparison between anti-VEGF and oral CAIs groups in RP-CME

Significant differences were observed between the two groups regarding changes in BCVA and CMT at months 3 and 6 compared to baseline, favoring the oral CAIs group (all *p* < 0.005). The BCVA change from baseline in the oral CAIs group improved by 0.15 logMAR at month 3 (*p* < 0.001) and by 0.12 logMAR at month 6 (*p* = 0.003) more than that in the anti-VEGF group. Additionally, the average CMT change from baseline in the oral CAIs group decreased by 96.01 μm at month 3 (*p* < 0.001) and by 83.40 μm at month 6 (*p* < 0.001) more than that in the anti-VEGF group. However, both the changes in BCVA and CMT showed no significant differences between the two groups at month 12 compared to baseline (*p* = 0.199 for BCVA, 0.239 for CMT) (Table 3; Supplementary Table S2, Figure 1).

**TABLE 3:** Intergroup comparisons of visual acuity, central macular thickness and intraocular pressure between oral CAIs and intravitreal anti-VEGF group using Generalized estimating equations.

Characteristics	BCVA (logMAR)		CMT (μm)		IOP (mmHg)	
	95%CI	*P*-value	95%CI	*P*-value	95%CI	*P*-value
Group (Oral CAIs vs. Anti-VEGF)						
Group*time (1 vs. 0)	[−0.040, 0.123]	0.317	[−59.743, 6.285]	0.113	[−0.380, 0.992]	0.382
Group*time (3 vs. 0)	[−0.215, −0.078]	**<0.001**	[−126.739, −65.278]	**<0.001**	[−0.288, 1.089]	0.254
Group*time (6 vs. 0)	[−0.191, −0.039]	**0.03**	[−116.861, −49.937]	**<0.001**	[−0.212, 1.137]	0.179
Group*time (12 vs. 0)	[−0.028, 0.134]	0.199	[−46.681, 11.652]	0.239	[−0.297, 1.024]	0.281

Group*time (1 vs. 0) represents comparing the change from baseline to 1 month after treatment within each group and then comparing these changes between the two groups and so forth.

CMT, central macular thickness; BCVA, best-corrected visual acuity; logMAR, logarithm of the minimum angle of resolution; IOP, intraocular pressure.

Bold values indicate statistical significance *P* < 0.05.

### Subgroups analysis in anti-VEGF and oral CAIs groups in RP-CME

In the oral CAIs subgroup, both the oral acetazolamide and methazolamide groups showed significant improvements in BCVA and reductions in CMT at months 1, 3, and 6 compared to baseline (all *p* < 0.001). However, by month 12, neither group had significant differences in BCVA and CMT compared to baseline (*p* > 0.05). Besides, the oral acetazolamide group demonstrated greater improvements in BCVA and reductions in CMT at month 3 (BCVA, *p* = 0.005; CMT, *p* = 0.037) and month 6 (BCVA, *p* = 0.015; CMT, *p* < 0.001) than the oral methazolamide group. In the anti-VEGF group, both the intravitreal ranibizumab and bevacizumab groups exhibited significant improvements in BCVA and reductions in CMT at months 1 and 3 compared to baseline (all *p* < 0.001). However, by months 6 and 12, neither group showed significant differences in BCVA and CMT compared to baseline (*p* > 0.05). There were no statistically significant differences in the improvements in BCVA and CMT between the oral ranibizumab and oral bevacizumab groups (*p* > 0.05) (Tables 4, 5; Supplementary Tables S3, S4; Figure 1).

**TABLE 4:** Comparisons of visual acuity and central macular thickness with baseline in two subgroups using Generalized estimating equations.

Group		Characteristics	BCVA (logMAR)	95%CI	*P*-value	CMT (μm)	95%CI	*P*-value
Anti-VEGF	Ranibizumab (n = 29)	Baseline	0.59 ± 0.19			434.70 ± 60.43		
		Month 1	0.46 ± 0.15	[−0.151, −0.102]	**<0.001**	374.45 ± 65.78	[−77.857, −42.626]	**<0.001**
		Month 3	0.55 ± 0.15	[−0.060, −0.013]	**0.002**	406.66 ± 70.57	[−43.477, −12.592]	**<0.001**
		Month 6	0.59 ± 0.16	[-0.022, 0.017]	0.807	437.03 ± 62.73	[−7.865, 12.555]	0.653
		Month 12	0.59 ± 0.16	[−0.051, 0.046]	0.922	433.45 ± 57.19	[−2.060, 19.578]	0.113
	Bevacizumab (n = 18)	Baseline	0.71 ± 0.30			426.44 ± 79.29		
		Month 1	0.56 ± 0.23	[−0.203, −0.105]	**<0.001**	363.61 ± 86.91	[−86.929, −38.738]	**<0.001**
		Month 3	0.62 ± 0.20	[−0.148, −0.033]	**0.002**	379.11 ± 58.16	[−73.579, −21.088]	**<0.001**
		Month 6	0.69 ± 0.26	[−0.069, 0.019]	0.270	416.95 ± 68.26	[−25.521, 6.521]	0.245
		Month 12	0.76 ± 0.26	[−0.034, 0.133]	0.244	423.50 ± 79.45	[−15.055, 9.166]	0.634
Oral CAIs	Methazolamide (n = 30)	Baseline	0.69 ± 0.23			434.40 ± 96.28		
		Month 1	0.56 ± 0.22	[−0.158, −0.100]	**<0.001**	359.47 ± 105.19	[−95.794, −54.073]	**<0.001**
		Month 3	0.48 ± 0.18	[−0.260, −0.156]	**<0.001**	319.07 ± 102.73	[−143.160, −87.506]	**<0.001**
		Month 6	0.56 ± 0.16	[−0.200, −0.069]	**<0.001**	391.10 ± 101.76	[−64.066, −22.534]	**<0.001**
		Month 12	0.71 ± 0.17	[−0.072,0.110]	0.683	431.73 ± 94.01	[−19.049, 13.716]	0.750
	Acetazolamide (n = 21)	Baseline	0.63 ± 0.23			417.76 ± 83.30		
		Month 1	0.51 ± 0.24	[−0.160, −0.090]	**<0.001**	320.86 ± 71.86	[−122.134, −71.676]	**<0.001**
		Month 3	0.36 ± 0.14	[−0.343, −0.207]	**<0.001**	273.00 ± 57.16	[−177.270, −112.254]	**<0.001**
		Month 6	0.44 ± 0.18	[−0.248, −0.137]	**<0.001**	281.43 ± 63.62	[−160.879, −111.787]	**<0.001**
		Month 12	0.70 ± 0.23	[−0.023, 0.156]	0.147	399.10 ± 57.93	[−37.646, 0.313]	0.054

CMT, central macular thickness; BCVA, best-corrected visual acuity; logMAR, logarithm of the minimum angle of resolution; IOP, intraocular pressure.

Bold values indicate statistical significance *P* < 0.05.

**TABLE 5:** Intergroup comparisons of visual acuity, central macular thickness and intraocular pressure between two subgroups using Generalized estimating equations.

	Characteristics	BCVA (logMAR)		CMT (μm)	
		95%CI	*P*-value	95%CI	*P*-value
Group (Bevacizumab vs. Ranibizumab)	Group*time (1 vs. 0)	[−0.020, 0.215]	0.103	[−56.397, 34.723]	0.641
	Group*time (3 vs. 0)	[−0.031, 0.174]	0.173	[−63.859, 8.771]	0.137
	Group*time (6 vs. 0)	[−0.029, 0.235]	0.126	[−58.069, 17.889]	0.300
	Group*time (12 vs. 0)	[0.049, 0.305]	**0.007**	[−61.065, 21.169]	0.342
Group (Acetazolamide vs. Methazolamide)	Group*time (1 vs. 0)	[−0.203, −0.105]	0.406	[−86.248, 9.029]	0.112
	Group*time (3 vs. 0)	[−0.148, −0.033]	**0.005**	[−89.373, −2.760]	**0.037**
	Group*time (6 vs. 0)	[−0.069, 0.019]	**0.015**	[−154.248, −65.095]	**<0.001**
	Group*time (12 vs. 0)	[−0.034, 0.133]	0.859	[−73.611, 8.334]	0.118

Group*time (1 vs. 0) represents comparing the change from baseline to 1 month after treatment within each group and then comparing these changes between the two groups and so forth.

CMT, central macular thickness; BCVA, best-corrected visual acuity; logMAR, logarithm of the minimum angle of resolution; IOP, intraocular pressure.

Bold values indicate statistical significance *P* < 0.05.

### Correlation analysis

In the correlation analysis, worse BCVA at month 12 is closely associated with older age (r = 0.202, *p* = 0.046), higher baseline CMT values (r = 0.353, *p* < 0.001), and poorer baseline BCVA (r = 0.579, *p* < 0.001). However, BCVA at month 12 have no correlation with sex, duration of RP and other RP genotypes (all *p* > 0.05) (Table 6).

**TABLE 6:** Correlation analysis of visual acuity at 12 months and other variables in all subjects.

Parameters	*r* Coefficients	*P*-value
Sex	0.125	0.220
Age	0.202	**0.046**
Duration of RP	0.142	0.162
CMT at baseline	0.353	**<0.001**
BCVA (logMAR) at baseline	0.579	**<0.001**
Autosomal dominant	−0.195	0.054
Autosomal recessive	0.151	0.317
X-linked	0.157 0.697	0.123

Bold values indicate statistical significance *P* < 0.05.

### Adverse events

In both groups, IOP remained within the normal range post-treatment. There was no significant difference in IOP in all time points (all *p* > 0.05) and between the two groups (*p* > 0.05) (Tables 2, 3). While patients in the anti-VEGF group did not experience significant side effects, multiple injections were required. Specifically, for the Ranibizumab group, the average number of injections was 3.7 ± 0.8, ranging from 1 to 6 injections, and for the Bevacizumab group, the average was 3.8 ± 0.5, ranging from 1 to 5 injections. In contrast, in the oral CAIs group, mild adverse effects were reported, including tingling sensations in 2 patients (3.9%), altered taste in 5 patients (9.8%), and gastrointestinal discomfort in 4 patients (7.8%). Edema recurrence rates were higher in the anti-VEGF group than in the oral CAIs group (51.1% vs. 29.4%, *p* = 0.048), with shorter mean recurrence times (2.5 ± 0.3 months vs. 4.2 ± 0.6 months, *p* < 0.001).

## Discussion

CME, a complication secondary to RP, results in severe visual damage with complicated and unclear pathogenesis. Various factors contribute to RP-CME, such as dysfunction in fluid pumping by retinal pigment epithelium (RPE) cells, breakdown of the blood-retinal barrier accompanied by mild inflammation, or the presence of anti-retinal antibodies. The epiretinal membrane may also trigger tractional CME. Several studies (Bakthavatchalam et al., 2018) have indicated that although many therapeutic interventions have been applied to treat RP-CME, there is currently no gold-standard therapy. Treatment response seems to be individualized, highlighting the need for tailored approaches to managing this condition.

Limited studies have compared the efficacy of intravitreal anti-VEGF agents to oral CAIs in managing RP-CME. Anti-VEGF agents, recognized for their vascular-protective and neuroprotective properties, offer benefits for vascular diseases, crucial for maintaining the function of photoreceptors and Müller cells, as well as improving microcirculation (Koc et al., 2023). Studies (Vinores et al., 1999) have indicated that VEGF release from RPE in patients with RP weakens the blood-retinal barrier and leads to macular edema. Anti-VEGF agents counteract the proliferation and cell migration stimulated by VEGF and the delocalization of tight junction proteins induced by VEGF. In a clinical trial conducted by Artunay et al. (2009), a single dose of intravitreal ranibizumab reduced CMT at 6 months post-injection, although the improvement in BCVA was not significant compared to the control group. Similarly, Strong et al. (2020) reported an 11% reduction in CMT at 12 months with serial intravitreal aflibercept (2 mg) therapy via loading dose (three injections) but found no significant improvement in BCVA. Bevacizumab was also identified as effective in reducing CMT and improving BCVA in 7 patients (Yuzbasioglu et al., 2009). These results were partly consistent with our study, which demonstrated the efficacy of anti-VEGF agents in reducing CMT. However, we observed a somewhat shorter duration of efficacy compared to other studies, with the efficacy of CMT lasting less than 3 months in our cohort. Moreover, the efficacy of BCVA improvement remained controversial across studies. This discrepancy may be due to differences in patient demographics, the severity of CME at baseline, or variations in treatment protocols. Future studies should explore these factors in more detail to better understand the underlying mechanisms. Despite no improvement in visual acuity among RP patients treated with anti-VEGF agents, our study and others have observed a significant resolution of macular edema. We speculate that chronic CME may still impact visual acuity even after macular edema resolution (Vinores et al., 1999).

CAIs, such as acetazolamide, methazolamide and topical 2% dorzolamide, represent the primary and preferred therapy for CME in eyes with RP (Shimokawa et al., 2020). Fishman et al. demonstrated an improvement in BCVA by more than 5 letters with oral methazolamide 50 mg twice a day for 3 weeks among all RP-CME patients (Fishman et al., 1989). Strong et al. (2019) have suggested visual acuity improvement with oral acetazolamide 500 mg/day for patients with RP-CME. Our study supports the efficacy of oral CAIs, especially acetazolamide, in reducing CMT and improving BCVA over 6 months, consistent with the findings of the aforementioned studies. Importantly, it demonstrated that oral CAIs provided superior anatomical and functional improvements compared to intravitreal anti-VEGF at 3 and 6 months post-treatment. This suggests that CAIs in RP-CME may operate through distinct therapeutic mechanisms, implicated in reducing retinal vascular leakage and enhancing active transport through the blood-retina barrier. This effect is attributed to the influence on the carbonic anhydrases isozyme IV, located at both apical and basal surfaces of the RPE. However, RP-CME patients with long-term oral CAIs had some adverse effects, including extremities tingling and altered taste sensation, which may limit its clinical use.

Of note, in our study, both BCVA and CMT at 12 months in patients with RP-CME had no significant difference compared with baseline, whether in the anti-VEGF group or the oral CAIs group. This lack of significant improvement may be attributed to the phenomenon of macular edema rebound observed in the patients and poor baseline condition such as high baseline CMT, older age and special genetype. Our study found that edema recurrence rate was high in both the anti-VEGF and oral CAIs groups. Besides, worse BCVA at month 12 was associated with higher baseline CMT, higher baseline BCVA, and older age in RP-CME patinets, indicating these factors might cause worse BCVA prognosis. Several studies reported that oxidative stress (Strong et al., 2020), and inflammation were significantly associated with the recurrence of CME and poor vision prognosis in RP-CME treatment. We speculated that reduced efficacy of intravitreal anti-VEGF injections and oral CAIs in the late stages of RP-CME can be attributed to several complex mechanisms, including changes in the underlying pathophysiology (Spaide, 2016), increased inflammation and fibrosis (Gao and Hollyfield, 1992; D’Amore, 1994), altered retinal microvascular and metabolic function (Todorova et al., 2021), retinal degeneration involving the loss of photoreceptors and retinal pigment epithelium cells (Swindle-Reilly et al., 2009; Dehghan et al., 2022), and potential resistance development (Massin et al., 2010) as RP progresses. These factors highlight the need for a multifaceted treatment approach in managing advanced RP-CME.

This study has several limitations. Firstly, although we reviewed nearly 15 years of case data, the sample size we included is small. However, the sample size was sufficient to achieve over 95% detection power, as confirmed by a sample size calculation using G*power (version 3.1.9.7). Retrospective studies inherently face limitations due to incomplete data and potential biases in patient selection. While we included only patients who completed 12 months of follow-up, indicating good adherence, adherence variability in a broader population could influence treatment outcomes. Moreover, our study involved four different treatment modalities—intravitreal injection of Ranibizumab and Bevacizumab, and oral Acetazolamide, and oral Methazolamide - each with potentially different response rates. This variability may affect the consistency of results and limits our ability to identify the most effective treatment. Future studies directly comparing these therapies are needed to clarify their efficacy. Additionally, our focus on anti-VEGF and oral CAIs excluded other potential treatments, limiting the scope of our findings. Research exploring a wider range of therapies could provide a more comprehensive understanding of optimal management strategies for RP-CME (Chen et al., 2022).

## Conclusion

In conclusion, our findings suggest that oral CAIs, especially acetazolamide, may be more effective than anti-VEGF therapy in improving visual function and restoring retinal anatomy in patients with RP-CME within 6 months. A worse BCVA prognosis is correlated with older age, higher baseline CMT, and poorer baseline visual acuity. Further clarification through prospective studies with larger sample sizes, longer follow-up periods and more therapy methods are necessary to better understand the optimal treatment approach for RP-CME patients.

## Data Availability

The original contributions presented in the study are included in the article/Supplementary Material. Further inquiries can be directed to the corresponding author.
