# Supplementary material for: Comparison of intravitreal anti-VEGF agents and oral carbonic anhydrase inhibitors in the treatment of cystoid macular edema secondary to retinitis pigmentosa
Source: Front Pharmacol. 2024 Dec 10;15:1477889. doi: 10.3389/fphar.2024.1477889 (PMC11683215; doi:10.3389/fphar.2024.1477889)
Supplement: Supplementary file 1 [file Table1.DOCX]

**Supplementary table 1** Intragroup comparisons of visual acuity, central macular thickness and intraocular pressure with baseline in two groups using Generalized estimating equations

| **Group** | **Characteristics** | **BCVA (logMAR)** | **Z value** | **P value** | **BCVA (logMAR)** | **Z value)** | **P value** | **IOP (mmHg)** | **Z value)** | **P value** |
| --- | --- | --- | --- | --- | --- | --- | --- | --- | --- | --- |
| **Anti-VEGF (n=47)** | Baseline | 0.64±0.24 |  |  | 431.53±67.55 |  |  | 12.75±1.47 |  |  |
|  | Month 1 | 0.50±0.19 | 119.625 | **＜0.001** | 370.30±73.85 | 70.806 | **＜0.001** | 12.73±1.49 | 0.116 | 0.734 |
|  | Month 3 | 0.58±0.17 | 16.973 | **＜0.001** | 396.11±66.82 | 24.224 | **＜0.001** | 12.77±1.49 | 0.099 | 0.752 |
|  | Month 6 | 0.62±0.21 | 1.064 | 0.302 | 429.34±64.92 | 0.230 | 0.631 | 13.71±1.40 | 0.332 | 0.564 |
|  | Month 12 | 0.65±0.22 | 0.597 | 0.440 | 435.81±66.48 | 1.022 | 0.312 | 12.74±1.36 | 0.004 | 0.950 |
| **Oral CAIs (n=51)** | Baseline | 0.67±0.23 |  |  | 427.55±90.67 |  |  | 13.09±1.94 |  |  |
|  | Month 1 | 0.54±0.23 | 125.352 | **＜0.001** | 343.57±94.09 | 101.357 | **＜0.001** | 13.03±1.99 | 0.630 | 0.427 |
|  | Month 3 | 0.43±0.18 | 117.478 | **＜0.001** | 300.10±89.17 | 134.799 | **＜0.001** | 13.17±2.00 | 1.582 | 0.208 |
|  | Month 6 | 0.51±0.18 | 46.261 | **＜0.001** | 345.94±102.94 | 62.509 | **＜0.001** | 13.17±2.02 | 1.268 | 0.260 |
|  | Month 12 | 0.71±0.19 | 1.324 | 0.250 | 418.29±82.05 | 2.074 | 0.150 | 13.11±1.97 | 0.070 | 0.791 |

CMT, central macular thickness; BCVA, best-corrected visual acuity; logMAR, logarithm of the minimum angle of resolution; IOP, intraocular pressure.

Data are presented as the means ± SD.

Bold values indicate statistical significance *P* < 0.05.

**Supplementary table 2** Intergroup comparisons of visual acuity, central macular thickness and intraocular pressure between oral CAIs and intravitreal anti-VEGF group using Generalized estimating equations

|  | **BCVA (logMAR)** | | | | **CMT (mm)** | | | | **IOP (mmHg)** | | | |
| --- | --- | --- | --- | --- | --- | --- | --- | --- | --- | --- | --- | --- |
| **Characteristics** | **B** | **Std. Error** | **Z value** | ***P* value** | **B** | **Std. Error** | **Z value** | ***P* value** | **B** | **Std. Error** | **Z value** | ***P* value** |
| Group (Oral CAIs vs. Anti-VEGF) |  |  |  |  |  |  |  |  |  |  |  |  |
| Group*time  (1 vs 0) | 0.042 | 0.042 | 1.001 | 0.317 | -26.729 | 16.844 | 2.518 | 0.113 | 0.306 | 0.350 | 0.764 | 0.382 |
| Group*time  (3 vs 0) | -0.146 | 0.035 | 17.721 | **< 0.001** | -96.008 | 15.679 | 37.495 | **< 0.001** | 0.400 | 0.351 | 1.300 | 0.254 |
| Group*time  (6 vs 0) | -0.115 | 0.039 | 8.799 | **0.003** | -83.399 | 17.073 | 23.862 | **< 0.001** | 0.462 | 0.344 | 1.803 | 0.179 |
| Group*time  (12 vs 0) | 0.053 | 0.041 | 1.652 | 0.199 | -17.514 | 14.881 | 1.385 | 0.239 | 0.363 | 0.337 | 1.162 | 0.281 |

Group*time (1 vs 0) represents comparing the change from baseline to 1 month after treatment within each group and then comparing these

changes between the two groups and so forth.

CMT, central macular thickness; BCVA, best-corrected visual acuity; logMAR, logarithm of the minimum angle of resolution; IOP, intraocular pressure.

Bold values indicate statistical significance *P* < 0.05.

**Supplementary table 3** Comparisons of visual acuity and central macular thickness with baseline in two subgroups using Generalized estimating equations

| **Group** |  | **Characteristics** | **BCVA (logMAR)** | **Z value** | **P value** | **CMT (mm)** | **Z value** | **P value** |
| --- | --- | --- | --- | --- | --- | --- | --- | --- |
| **Anti-VEGF** | **Ranibizumab (n = 29)** | Baseline | 0.59±0.19 |  |  | 434.70±60.43 |  |  |
|  |  | Month 1 | 0.46±0.15 | 101.265 | **＜0.001** | 374.45±65.78 | 44.924 | **＜0.001** |
|  |  | Month 3 | 0.55±0.15 | 9.648 | **0.002** | 406.66±70.57 | 12.661 | **＜0.001** |
|  |  | Month 6 | 0.59±0.16 | 0.060 | 0.807 | 437.03±62.73 | 0.203 | 0.653 |
|  |  | Month 12 | 0.59±0.16 | 0.010 | 0.922 | 433.45±57.19 | 2.518 | 0.113 |
|  | **Bevacizumab**  **(n = 18)** | Baseline | 0.71±0.30 |  |  | 426.44±79.29 |  |  |
|  |  | Month 1 | 0.56±0.23 | 37.460 | **＜0.001** | 363.61±86.91 | 26.122 | **＜0.001** |
|  |  | Month 3 | 0.62±0.20 | 9.575 | **0.002** | 379.11±58.16 | 12.494 | **＜0.001** |
|  |  | Month 6 | 0.69±0.26 | 1.215 | 0.270 | 416.95±68.26 | 1.351 | 0.245 |
|  |  | Month 12 | 0.76±0.26 | 1.355 | 0.244 | 423.50±79.45 | 0.227 | 0.634 |
| **Oral CAIs** | **Methazolamide**  **(n = 30)** | Baseline | 0.69±0.23 |  |  | 434.40±96.28 |  |  |
|  |  | Month 1 | 0.56±0.22 | 76.698 | **＜0.001** | 359.47±105.19 | 49.568 | **＜0.001** |
|  |  | Month 3 | 0.48±0.18 | 60.851 | **＜0.001** | 319.07±102.73 | 65.990 | **＜0.001** |
|  |  | Month 6 | 0.56±0.16 | 16.015 | **＜0.001** | 391.10±101.76 | 16.702 | **＜0.001** |
|  |  | Month 12 | 0.71±0.17 | 0.166 | 0.683 | 431.73±94.01 | 0.102 | 0.750 |
|  | **Acetazolamide**  **(n = 21)** | Baseline | 0.63±0.23 |  |  | 417.76±83.30 |  |  |
|  |  | Month 1 | 0.51±0.24 | 48.849 | **＜0.001** | 320.86±71.86 | 56.675 | **＜0.001** |
|  |  | Month 3 | 0.36±0.14 | 62.548 | **＜0.001** | 273.00±57.16 | 76.178 | **＜0.001** |
|  |  | Month 6 | 0.44±0.18 | 46.227 | **＜0.001** | 281.43±63.62 | 118.504 | **＜0.001** |
|  |  | Month 12 | 0.70±0.23 | 2.103 | 0.147 | 399.10±57.93 | 3.716 | 0.054 |

CMT, central macular thickness; BCVA, best-corrected visual acuity; logMAR, logarithm of the minimum angle of resolution; IOP, intraocular pressure.

Bold values indicate statistical significance *P* < 0.05.

**Supplementary table 4** Intergroup comparisons of visual acuity, central macular thickness and intraocular pressure between two subgroups using Generalized estimating equations

|  |  | **BCVA (logMAR)** | | | | **CMT (mm)** | | | |
| --- | --- | --- | --- | --- | --- | --- | --- | --- | --- |
|  | **Characteristics** | **B** | **Std. Error** | **Z value** | ***P* value** | **B** | **Std. Error** | **Z value** | ***P* value** |
| Group (Bevacizumab vs. Ranibizumab ) | Group*time  (1 vs 0) | 0.098 | 0.060 | 2.653 | 0.103 | -10.837 | 23.245 | 0.217 | 0.641 |
|  | Group*time  (3 vs 0) | 0.071 | 0.052 | 1.855 | 0.173 | -27.544 | 18.528 | 2.210 | 0.137 |
|  | Group*time  (6 vs 0) | 0.103 | 0.067 | 2.340 | 0.126 | -20.090 | 19.377 | 1.075 | 0.300 |
|  | Group*time  (12 vs 0) | 0.177 | 0.065 | 7.382 | **0.007** | -19.948 | 20.978 | 0.904 | 0.342 |
| Group  (Acetazolamide  vs. Methazolamide) | Group*time  (1 vs 0) | -0.053 | 0.064 | 0.692 | 0.406 | -38.610 | 24.306 | 2.523 | 0.112 |
|  | Group*time  (3 vs 0) | -0.125 | 0.045 | 7.742 | **0.005** | -46.067 | 22.096 | 4.347 | **0.037** |
|  | Group*time  (6 vs 0) | -0.116 | 0.048 | 5.872 | **0.015** | -109.671 | 22.743 | 23.253 | **＜0.001** |
|  | Group*time  (12 vs 0) | -0.010 | 0.057 | 0.032 | 0.859 | -32.638 | 20.905 | 2.438 | 0.118 |

Group*time (1 vs 0) represents comparing the change from baseline to 1 month after treatment within each group and then comparing these

changes between the two groups and so forth.

CMT, central macular thickness; BCVA, best-corrected visual acuity; logMAR, logarithm of the minimum angle of resolution; IOP, intraocular pressure.

Bold values indicate statistical significance *P* < 0.05.
